# Supplementary material for: The G protein-first activation mechanism of opioid receptors by Gi protein and agonists
Source: QRB Discov. 2021 Aug 5;2:e9. doi: 10.1017/qrd.2021.7 (PMC10392629; doi:10.1017/qrd.2021.7)
Supplement: Supplementary file 1 [file S2633289221000077sup001.docx]

**Supplementary Information**

**G protein-first activation mechanism of opioid receptors by Gi protein and agonists**

Amirhossein Mafi, Soo-Kyung Kim, and William A. Goddard III*^*^*

*Materials and Process Simulation Center (139-74)*

*California Institute of Technology, Pasadena, California 91125, United States*

*E-mail: [wag@caltech.edu](mailto:wag@caltech.edu); ORCID:0000-0003-0097-5716

Soo-Kyung Kim ORCID: 0000-0002-4498-5441

Amirhossein Mafi ORCID: 0000-0002-8366-6785

**Contents**

1. **Details of MD and metaMD simulations**

- Mouse-μOR in complex with Gi
- Gαi preparation.
- Modeling of fully active human- μOR -morphine-Gi protein.
- Modeling of fully active human δOR-DPI-287-Gi protein.
- MD simulation of fully active human opioid receptors-Gi protein complex.
- Refinement of Cryo-EM mouse μOR-DAMGO-Gi protein.
- Modeling of pre-activated human-μOR-Gi (GDP) complex.
- Activation of pre-activated state of μOR-Gi protein by morphine (transition from Σ3’ to Σ4’*).
- Optimization of μOR-morphine: Initiation of activation by Ligand-First mechanism of activation.
- Optimization of μOR-agonists derived from the fully active crystal and Cryo-EM structures.
- Modeling of pre-activated human-κOR-Gi (GDP) complex.
- Activation of pre-activated state of κOR -Gi protein by MP1104 (transition from Σ3’ to Σ4’*).
- Optimization of κOR-MP1104: Initiation of activation by Ligand-First mechanism of activation.
- Modeling of pre-activated human-δOR-Gi (GDP) complex.
- Activation of pre-activated state of δOR-Gi protein by DPI-287 (transition from Σ3’ to Σ4’*).
- Optimization of δOR-DPI-287 (S1 state): Initiation of activation by Ligand-First mechanism of activation.
- Force fields and algorithms for free energy calculations.

1. **Figures**

- Fig. S1. Evaluation of the strength for the anchors between Gi protein and each of three ICLs of mouse μOR.
- Fig. S2. Structure of the mouse μOR–Gi Protein complex derived from MD simulations.
- Fig. S3. Gi protein binds the human μOR by forming ionic anchors to ICL1, ICL2, and the cytoplasmic end of TM6, despite the choice of force fields.
- Fig. S4. Evaluation of the energetics relevant to the formation of pre-activated state between Gi protein and μOR.
- Fig. S5. Evaluation of the energetics relevant to the formation of pre-activated state between the Gα-α5 peptide and μOR.
- Fig. S6. Evaluation of the energetics relevant to the formation of pre-activated state between Gi protein and either of κOR or δOR.
- Fig. S7. A candidate structure through the formation of the Gi protein-δOR pre-activated complex.
- Fig. S8. The Orthosteric binding pocket of agonist in the Σ3’ of opioid receptors-Gi protein complex.
- Fig. S9. Agonists cannot not stabilize the active conformation of μOR.
- Fig. S10. The energetically optimized S1 state of κOR-MP1104 and δOR-DPI-287.
- Fig. S11. Gαi subunit interactions with GDP in pre-activated state (Σ2).
- Fig. S12. Evaluation of the RMSD for: A) Gαi-α5 peptide, B) μOR, C) μOR- Gαi-α5 peptide during the formation of pre-activated state between the Gα-α5 peptide and μOR.
- Fig. S13. The second replicate of metaMD calculations to assess the formation of pre-activated complex between Gi protein and human μOR prior to ligand binding.
- Fig. S14. Binding of agonist to μOR cannot open the TM3-6 hydrogen bond in the absence of Gi protein.
- Fig. S15. Comparisons of the cytoplasmic region of the inactive state and the pre-activated state for (A) μOR, (B) κOR, and (C) δOR. All RMSDs were calculated for the Cα atoms on the 7TM core.
- Fig*.* S16. Agonist binding is the main driver of activation for the μOR and Gi protein.
- Fig. S17. Activation of the Gi protein is induced by agonist binding to the opioid receptors-Gi Protein complex.

**1. Details of MD and metaMD simulations**

**Mouse-μOR in complex with Gi****.** Some atoms were not fully resolved or missing in the Cryo-EM structure(*1*):

- residues L5, E28, E43, I55, E207, N241, K270, E275, K279, K280, I285, C305, E308, E318, T327, D337, D350 in Gαi,
- residues K23, C25, S31, R46, E130, R214, M217, N237, C271, N237, C271, K301, D312, C317 in Gβ,
- residues D26, D48, S57, E58, in Gγ, and
- residues M65^1.29^, V66^1.30^, T67^1.31^, I69^1.33^, M72^1.36^, L74^1.38^, S76^1.40^, I77^1.41^, V80^1.44^, V81^1.45^, V94^1.58^, R95^1.59^, K100, D114^2.50^, S119^2.55^, S125^2.61^, L129^2.65^, T132, N137^3.22^, I138^3.23^, K185^4.43^, I186^4.44^, I193^4.51^, M203^4.61^ , K209, Q212, I215, F221, N230^5.37^, L246^5.52^, V291^6.46^, T294^6.49^, K303^6.58^, T307, F313^7.29^, S317^7.33^, F343^8.50^, K344^8.51^, R345^8.52^ in the μOR,

We added these missing atoms using Swiss-pdbviewer(*2*) where during the process we also reconstructed the sidechains. The following residues played important roles in forming the anchors:

E28^Gαi-αN helix^, E308^Gαi^, E318^Gαi^, D312^Gβ^, and K100^ICL1^

**Gαi preparation.**The entire amino acid residues in the AH domain (residues 56 to 181 and 234 to 240) in the Gαi subunit (PDB ID: 6DDF)(*1*) were all missing in the Cryo-EM structure. These missing residues were added by superimposing the Gαi to the one resolved in the Cryo-EM Gi protein-rhodopsin complex (PDB ID: 6CMO). Then, the newly added connections between residues in Ras-like and AH domains was energetically minimized using conjugated gradients for 500 steps. During the energy minimization process, all heavy atoms in the Ras-like domain were restrained with a strong harmonic force constant of ~24 kcal.mol^-1^A^-2^ to avoid any undesirable changes in the structure of Gαi.

**Modeling of** **fully active human- μOR -morphine-Gi protein.** To predict the active conformation of human-μOR, we used the active conformation of mouse-μOR [PDB ID: 5C1M](*3*) as a template for GEnSeMBLE predictions(*4*). The human and mouse μ opioid receptors shares 94% sequence identity for the entire protein sequence and shares 99% sequence identity for the residues resolved by the crystallography. For the morphine binding site, there is 100% sequence identity, allowing us to use the mouse structure as the template for our modeling. Using the predicted active conformation of human-μOR, we inserted morphine to orthosteric binding pocket using the DarwinDock procedure(*5*). The final optimized pharmacophore obtained from MD simulation (Figure 3J) is consistent with previous observations of morphinan scaffold ligands in complex with the μOR.(*1*, *3*, *6*) Subsequently, we aligned human μOR-bound morphine to the optimized mouse-μOR-Gi protein-DAMGO complex. After alignment, the mouse μOR was substituted by the predicated human μOR-bound morphine.

**Modeling of fully active human δOR-DPI-287-Gi protein.** Ten residues were not fully resolved in the crystal structure (*7*) of human-δOR bound to DPI-287 agonist (PDB ID:6PT3) including R76, K79, K81, K155, K250, E251, K252, R254, R291, and R327. Of these residues, we find that K79 and K250 involve ionic anchors with Gi protein (Figure 4H& 4J). We added these missing atoms/side chains using Swiss-Pdbviewer(*2*), where during the process we also reconstructed the side chains. Then, we removed the whole chain B and T4L protein from the chain A of crystal structure. Subsequently, we aligned human δOR-bound DPI-287 to the optimized mouse-μOR-Gi protein-DAMGO complex. After alignment, the mouse μOR was substituted by the human δOR-bound DPI-287.

**MD simulation of fully active human opioid receptors-Gi protein complex.** We immersed the human μOR-morphine-Gi protein and δOR- DPI-287-Gi protein complexes into membrane bilayer, composed of 277 palmitoyl-oleoyl-phosphatidylcholine POPC molecules. We used our pre-equilibrated somatostatin receptor-5-Gi complex(*8*) to superimpose the opioid receptors to the somatostatin receptor-5 in order to place human μOR/δOR into the POPC membrane. Subsequently, we solvated the resulting construct by water, and ions which led to a simulation box of 100×100×147Å^3^ with ~151,000 atoms. Then, to optimize the human μOR-morphine- Gi protein complex, we carried out three replicates of ~1μs MD simulations using Amber14(*9*), Charmm36m(*10*), and OPLS(*11*) force fields. However, to equilibrate the human δOR- DPI-287-Gi complex, we carried out a ~300ns MD simulation using Charmm36m (*10*) force field.

To optimize each replicate of human μOR-morphine-Gi, we carried out 5000 steps of energy minimization using the steepest descent algorithm. Subsequently, we carried out a ~1ns MD simulation where we placed positional restraints on the heavy atoms with a force constant of 9.6 kcal.mol^-1^ Å^-2^. In addition, we restrained the z-coordinate of the headgroups of POPC inside the membrane with a force constant of ~2.4 kcal.mol^-1^Å^-2^ so that the POPC molecules can move freely along the xy-plane to find their appropriate packing. Throughout this calculation, the restraints on the protein, morphine, and POPC were gradually reduced to 0 kcal.mol^-1^Å^-2^, which prepared the construct for the further relaxation. Then, we removed all restraints and performed ~150 ns MD simulation in isothermal-isobaric ensemble to relax the complex. Eventually, we carried out a ~1μs MD simulation on the complex to further relax the system and for our analysis. Figure 3 and Figure S3 were made from the results of this calculation.

To equilibrate the human μOR-DPI-287-Gi protein, we first implemented 5000 steps of energy minimization using the steepest descent algorithm. Subsequently, we carried out a ~5ns MD simulation where we placed positional restraints on the heavy atoms with a force constant of 9.6 kcal.mol^-1^ Å^-2^. In addition, we restrained the z-coordinate of the headgroups of POPC inside the membrane with a force constant of ~2.4 kcal.mol^-1^Å^-2^. Throughout this calculation, the restraints on the protein, DPI-287, and POPC were gradually reduced to 0 kcal.mol^-1^Å^-2^, which prepared the construct for the further relaxation. Then, we removed all restraints and performed ~300 ns MD simulation in isothermal-isobaric ensemble to relax the complex. Figure 4 was made from the results of this calculation.

For these simulations, the temperature was maintained at 310K and the pressure was controlled at 1 bar. The semi-isotropic pressure coupling was used during this calculation. The Lennard-Jones cutoff radius was 12 Å, where, the interaction was smoothly shifted to 0 after 12 Å. Periodic boundary conditions were applied to all three directions. The particle mesh Ewald (*12*) with a real cutoff radius of 12Å and a grid spacing of 1.6 Å was used to calculate the long-range columbic interactions. The compressibility of 4.5 ×10^-5^ bar^-1^ was used in the xy- plane and also the z-axis, to relax the box volume.

**Refinement of Cryo-EM mouse μOR-DAMGO-Gi protein.** To optimize the μOR-DAMGO-Gi protein complex, we carried out 2000 steps of energy minimization using the steepest descent algorithm. Subsequently, we carried out a ~2ns MD simulation where we placed positional restraints on the heavy atoms with a force constant of 9.6 kcal.mol^-1^ Å^-2^. In addition, we restrained the z-coordinate of the headgroups of POPC inside the membrane with a force constant of ~2.4 kcal.mol^-1^Å^-2^. Throughout this calculation, the restraints on the protein, DAMGO, and POPC were gradually reduced to 0 kcal.mol^-1^Å^-2^, which prepared the construct for the further relaxation. However, to ensure that the shape of protein from cryo-EM is not disturbed while the newly added residues are relaxed, we kept those positional restraints on the protein backbone atoms from the Cryo-EM structure (excluding the newly added residues). Then, we performed ~450ns MD simulation using Amber14(*9*), where we kept positional restraints on the backbone atoms with a force constant of 9.6 kcal.mol^-1^.Å^-2^. We used the results of the last 100ns frames to calculate the density map using GROmaps(*13*) for those atoms resolved by Cryo-EM structure of μOR-DAMGO-Gi (EMDB-7869)(*1*) in order to have fair comparisons. Finally, we used ChimeraX(*14*) for alignment of density maps obtained from MD simulation and that from Cryo-EM. Figure 2 & Figure S2 were made from the results of these calculations and analysis.

We assessed the energetics of all three salt bridge anchors between each of three ICLs and Gi protein with metaMD simulations. To do this, we used the final equilibrated structure of mouse μOR-Gi-DAMGO. Keeping the restraints on the protein backbone atoms, we carried out 2 sets of metaMD calculations using Amber14(*9*) on the following collective variables:

1. K98^ICL1^(CZ)-D312^Gβi^ (CG) and D177^ICL2^(CG)-R32^G^*^α^*^i^ (CZ)
2. K271^6.26^(CZ)-E318^G^*^α^*^i^ (CD) and R263^ICL3^(CZ) and E318^G^*^α^*^i^ (CD)

We imposed the upper walls (a force constant ~120 kcal.mol^-1^Å^-2^ at a distance of 9Å) on each of collective variables and used a time step of 2.5fs to expedite the process. The final free energy profiles were shown in Figure 2 & S1. For these two calculations, the metaMD bias was deposited with a Gaussian width of 0.5Å, an initial Gaussian amplitude of 0.48 kcal/mol, a deposition period of 1.0 ps and a bias factor of 15.

**Modeling of pre-activated human-μOR-Gi (GDP) complex.** We started from the crystal structure(*15*) of heterothermic Gi protein bound with GDP (PDB ID: 1GP2), and added missing side chains using Swiss-pdbviewer(*2*). Then, we extended the Gαi-αN and Gαi-α5 helices after superimposing to the Gαi subunit of the cryo-EM structure (Pdb ID: 6DDF) Eventually, the Gαi-α5 helix was optimized by placing intra-helical hydrogen bond restraints with a force constant of ~1.4 kcal.mol^-1^Å^-2^. The distance between N (i+4) and C(i) atoms of the residues in all α-helices were restrained at a distance of 4.1 Å with a force constant of ~1.4 kcal.mol^-1^Å^-2^. Then, to predict an inactive structure for the human-μOR, we used the crystal structure of μOR [PDB ID: 4DKL] (*6*) as a template for GEnSeMBLE predictions(*4*). Finally, each modeled μOR and Gi protein were separately aligned to the μOR and Gi protein of mouse active structure, respectively in order to build a pre-coupled state in which the Gi protein was placed in close proximity of ICLs of μOR, enabling it to make salt bridge contacts to μOR.

Placing the inactive human-μOR in contact with tight Gi-bound GDP into the POPC membrane, we performed the following steps to minimize the structure:

We carried out 5000 steps of steepest descent energy minimization to relax the lipid packing around the protein construct and to relax the protein complex as well. During this process, the positions of all heavy atoms of proteins and GDP were restrained with a force constant of ~9.6 kcal.mol^-1^Å^-2^. For POPC, we used position restraints with a force constant of ~2.4 kcal.mol^-1^Å^-2^ on just the z-coordinate of heavy atoms so that the POPC could move freely along the xy- plane to find the appropriate packing around the protein. Subsequently, we carried out a ~9ns MD simulation where we placed positional restraints on the heavy atoms with a force constant of 9.6 kcal.mol^-1^ Å^-2^. In addition, we restrained the z-coordinate of the headgroups of POPC inside the membrane with a force constant of ~2.4 kcal.mol^-1^Å^-2^. Throughout this calculation, the restraints on the protein, GDP, and POPC were gradually reduced to 0 kcal.mol^-1^Å^-2^, which prepared the construct for the further energy minimization. Then, we minimized the complex by performing a ~900ns metaMD simulation using Amber14(*9*) in which we applied biased forces on two key interactions;

1. the salt bridge between the carboxylate group of F354(C) and R167^3.50^(CZ) and
2. the hydrophobic interaction between F354 (Cα) -R167^3.50^ (Cα).

We imposed upper walls on our collective variables with force constants of ~119.5 kcal.mol^-1^Å^-2^ at distances of 12 and 10Å, respectively. We placed another harmonic wall on the interaction between R167^3.50^ (CZ)- T281^6.34^ (OG1), with a force constant of ~119.5 kcal.mol^-1^Å^-2^ at a distance of 6.5Å to expedite the sampling process.

To test if the salt bridge between F354(C)-R167^3.50^(CZ) weakens the TM3-TM6 coupling, we evaluated the energetics for opening the polar interaction between R167^3.50^ (CZ)- T281^6.34^ (OG1) while a salt-bridge interaction is already formed between F354(F-1)-R167^3.50^. To calculate the free energy, we applied metaMD bias forces on two important interactions:

1. the polar interaction between T281^6.34^(OG1)-R167^3.50^(CZ) and
2. the hydrophobic interaction between T281^6.34^(Cα)-R167^3.50^(Cα)

where we placed a restraint with a force constant of ~1.9 kcal.mol^-1^Å^-2^ on the salt bridge between F354-R167^3.50^. We also imposed harmonic upper walls on our collective variables with force constants of ~119.5 kcal.mol^-1^Å^-2^ at distances of 9 and 11Å, respectively. For these calculations, we placed intra-helical hydrogen bond restraints with force constants of ~1.4 kcal.mol^-1^Å^-2^ on the protein. The bias was imposed with a Gaussian width of 0.5 Å, an initial Gaussian amplitude of 0.48 kcal/mol, a deposition period of 1.0 ps and a bias factor of 15.

To exclude the possibility that the specific rigid-body orientation of Gi protein led to emergence of pre-coupled state between μOR -Gi, we independently modeled a pre-coupled state in which we included only the Gαi-α5 peptide (the last 21 residues: ^334^F-F^354^) and placed it in close proximity to the inactive μOR. Then, to follow our molecular mechanism of G Protien-First mechanism of activation, we performed a ~1μs metaMD free energy calculation using Amber14(*9*) force field where, we evaluated the energetics relevant to following interactions:

1. a salt bridge between the carboxylate group of F354 (C) and R176^3.50^(CZ)
2. a polar interaction between R167^3.50^(CZ)-T281^6.34^ (OG1)
3. a hydrophobic interaction between F354 (Cα) -R167^3.50^ (Cα).

To expedite this calculation and limit the sampling phase space, we imposed upper walls on all collective variables with force constants of ~24 kcal.mol^-1^Å^-2^ at a distance of 10 Å. For this calculation, intra-helical hydrogen bond restraints with force constants of ~1.4 kcal.mol^-1^Å^-2^ were placed on the proteins. The metaMD bias was deposited with a Gaussian width of 0.5Å, an initial Gaussian amplitude of 0.48 kcal/mol, a deposition period of 1.0 ps and a bias factor of 15. The results of these calculations were presented in Figure 4A-I & Figure S4-5.

**Activation of pre-activated state of μOR-Gi protein by morphine (transition from Σ3’ to Σ4’*).** To insert morphine to the pre-activated state (Σ2), we exploited our fully optimized morphine conformation in the orthosteric binding pocket of the human μOR in its fully active state (Σ4’ state). Thus, we superimposed the Σ2 state to the fully active human μOR (Σ4’). To locate and optimize the interactions between human-μOR and morphine, we performed 10ns (500 cycles) of simulated annealing, in which the system was first heated from 25 to 600 K over 20ps with a sequence of 25, 100, 310, 450, 600K and then sharply cooled back to 310K over 30 ps. In this calculation, we placed harmonic restraints on backbone atoms with a force constant of ~9.6 kcal.mol^-1^Å^-2^, while the side chains were free to find the optimum conformation. The resulting binding pocket of Σ3’ state was shown in Figure S8.

We evaluated the energetics relevant to opening of the Gαi induced by the morphine binding to the pre-activated state. we performed a ~400ns metaMD using Amber14(*9*) force field to apply bias forces on three key variables:

1. Opening the distance between TM3 (center of mass of Cα atoms of last 11 residues in the cytoplasmic region) and TM6 (center of mass of Cα atoms of last 11 residues in the cytoplasmic region),
2. Closing the distance between TM5 (center of mass of Cα atoms of last 11 residues in the cytoplasmic region) and TM6, and
3. Opening the Gαi from AH (the center of mass of the Cα atoms for the residues 147-181) and Ras-like domain (the center of mass of the Cα atoms for the residues 42-59), which define the GDP biding site.

During this simulation, we imposed upper walls on our collective variables with force constants of ~24 kcal.mol^-1^Å^-2^ at distances of 17.5, 13.5, and 26Å, respectively to expedite the sampling process. We also placed a restraint with a force constant of ~1.2 kcal.mol^-1^Å^-2^ on the typical salt bridge between D149^3.32^ and the protonated N atom of morphine. In addition, we limited the sampling space by applying lower walls on our collective variables with force constants of ~24 kcal.mol^-1^Å^-2^ at distances of 12, 7, and 15Å, respectively. During this calculation, intra-helical hydrogen restraints with force constants of ~1.4 kcal.mol^-1^Å^-2^ were placed on the proteins. Also, we kept the anchors by placing harmonic restraints with a force constant ~0.6 kcal.mol^-1^Å^-2^.

Here, the metaMD bias the bias was deposited with a Gaussian width of 1 Å, an initial Gaussian amplitude of 0.72 kcal/mol, a deposition period of 1.0 ps and a bias factor of 25. The results of these calculations were presented in Figure 6.

**Optimization** **of μOR-morphine: Initiation of activation by Ligand-First mechanism of activation.** To insert morphine to the inactive state (Σ0) of μOR**,** we exploited our fully optimized morphine conformation in the orthosteric binding pocket of the human μOR in its fully active state (Σ4’ state). Thus, we superimposed the Σ0 state to the fully active human μOR (Σ4’). To locate and optimize the interactions between human-μOR and morphine, we performed 10ns (500 cycles) of simulated annealing, in which the system was first heated from 25 to 600 K over 20ps with a sequence of 25, 100, 310, 450, 600K and then sharply cooled back to 310K over 30 ps. In this calculation, we placed harmonic restraints on backbone atoms with a force constant of ~9.6 kcal.mol^-1^Å^-2^, while the side chains were free to find the optimum conformation. The resulting binding pocket of Σ3’ state was shown in Figure 7A.

To determine whether morphine can induce a large expansion in the cytoplasmic region of μOR, we carried out a ~910ns metaMD simulation Amber14(*9*) force field where we evaluate the energetics relevant to repositioning of TM6. Thus, we applied the bias forces on:

1. the distance between TM3 [the center of mass of Cα for residues 161-172] and TM6 [the center of mass of Cα for residues 274-285],
2. the distance between TM6 and TM7 [the center of mass of Cα for residues 330-341].
3. the distance between TM6 and TM5 [the center of mass of Cα for residues 253-264].

To expedite these calculations and limit the sampling phase space, we imposed upper and lower walls with force constant of ~24.0 kcal.mol^-1^Å^-2^ such that the distances: TM3-TM6, TM5-TM6, and TM6-TM7 vary over a range of [9.5-18.5Å], [9-15.0Å], [18.5-24.5Å], respectively.

During these calculations, we placed the inter-helical hydrogen bonds restraints, as mentioned above with a force constant of ~1.4 kcal.mol^-1^Å^-2^, to relax the protein structure while ensuring that the α-helical structures are stable and intact over the course of simulation. However, we applied reduced force constants of 0.24 kcal.mol^-1^Å^-2^ on TM6 (residues 274-285) to ensure that TM6 is flexible enough for its large outward movement.

The metaMD bias was deposited with a Gaussian width of 1.0Å, an initial Gaussian amplitude of 0.72 kcal/mol, a deposition period of 1.0 ps and a bias factor of 25.

Subsequently to examine if morphine binding induces the polar interaction between R167^3.50^-T281^6.34^ to break open, we separately performed a ~1.5μs metaMD simulation using Amber14(*9*) to assess the energetics relevant to following hydrogen bonds:

1. T281^6.34^(OG1)-R167^3.50^(NE)
2. T281^6.34^(OG1)-R167^3.50^(NH1)
3. T281^6.34^(OG1)-R167^3.50^(NH2)

To expedite this process, we imposed upper walls on our collective variables with force constants of ~24 kcal.mol^-1^Å^-2^ at distances of 8.5Å. Also, we placed a set of harmonic restraints on the inter-helical hydrogen bonds in order to relax the protein structure while ensuring that the α-helical structures are stable and intact over the course of simulation. The distance between N (i+4) and C (i) atoms of the residues in all α-helices were restrained at a distance of 4.1 Å with a force constant of ~1.2 kcal.mol^-1^Å^-2^. We also placed a restraint with a force constant of ~1.2 kcal.mol^-1^Å^-2^ at a distance of 3 Å of the typical salt bridge between D149^3.32^ and the protonated N atom of morphine. The metaMD bias was deposited with a Gaussian width of 0.5Å, an initial Gaussian amplitude of 0.29 kcal/mol, a deposition period of 1.0 ps and a bias factor of 12. The structures corresponding to the minimum of free energy were shown in Figure 7A. We find that the minimized μOR-bound morphine adopts a closed cytoplasmic packing that closely matches the crystallographic inactive μOR (Figure 7B). The results of these calculations were presented in Figure 7A-E & Figure S9.

**Optimization of μOR-agonists derived from the fully active crystal and Cryo-EM structures.** To model the μOR-bound DAMGO, we started with our optimized mouse μOR-DAMGO-Gi protein complex and removed the Gi protein from the structure, which was basically resolved by Cryo-EM(*1*). Subsequently, we immersed the resulting μOR-DAMGO complex into a 277 POPC membrane bilayer and solvated it with water and ions which led to a simulation box of 100×100×77 Å^3^, consisted of 80,000 atoms. We carried out 5000 steps of steepest descent energy minimization to relax the lipid around the protein construct and to relax the protein complex as well. During this process, the positions of all heavy atoms of proteins and DAMGO were restrained with a force constant of ~9.6 kcal.mol^-1^Å^-2^. For POPC, we used position restraints with a force constant of ~2.4 kcal.mol^-1^Å^-2^ on just the z-coordinate of heavy atoms so that the POPC could move freely along the xy- plane to find the appropriate packing around the protein. Subsequently, we carried out a ~2ns MD simulation where we placed positional restraints on the heavy atoms with a force constant of 9.6 kcal.mol^-1^ Å^-2^. In addition, we restrained the z-coordinate of the headgroups of POPC inside the membrane with a force constant of ~2.4 kcal.mol^-1^Å^-2^. Throughout this calculation, the restraints on the protein, DAMGO, and POPC were gradually reduced to 0 kcal.mol^-1^Å^-2^, which prepared the construct for the further energy minimization.

To determine whether DAMGO can stabilize the widely open cytoplasmic region of mouse μOR, we carried out a ~600ns metaMD simulation using Amber14(*9*) where we evaluate the energetics relevant to repositioning of TM6. Thus, we applied the bias forces on:

1. the distance between TM3 [the center of mass of Cα for residues 159-170] and TM6 [the center of mass of Cα for residues 272-283],
2. the distance between TM6 and TM7 [the center of mass of Cα for residues 328-339].

To expedite these calculations and limit the sampling phase space, we imposed upper and lower walls with force constant of ~24.0 kcal.mol^-1^Å^-2^ such that the distances: TM3-TM6, TM5-TM6, and TM6-TM7 vary over a range of [10.0-16.0Å],[<11Å], and [13.5-22.5Å], respectively.

During these calculations, we placed the inter-helical hydrogen bonds restraints, as mentioned above with a force constant of ~1.4 kcal.mol^-1^Å^-2^, to relax the protein structure while ensuring that the α-helical structures are stable and intact over the course of simulation.

The metaMD bias was deposited with a Gaussian width of 1.0Å, an initial Gaussian amplitude of 0.72 kcal/mol, a deposition period of 1.0 ps and a bias factor of 25.

Subsequently to examine if DMAGO binding induces the polar interaction between R165^3.50^-T279^6.34^ to break open, we separately performed a ~860ns metaMD simulation using Amber14(*9*) force field to assess the energetics relevant to following hydrogen bonds:

1. T279^6.34^(OG1)-R165^3.50^(NE)
2. T279^6.34^(OG1)-R165^3.50^(NH1)
3. T279^6.34^(OG1)-R165^3.50^(NH2)

To expedite this process, we imposed upper walls on our collective variables with force constants of ~24 kcal.mol^-1^Å^-2^ at distances of 8.5 Å. Also, we placed a set of harmonic restraints on the inter-helical hydrogen bonds in order to relax the protein structure while ensuring that the α-helical structures are stable and intact over the course of simulation. The distance between N (i+4) and C (i) atoms of the residues in all α-helices were restrained at a distance of 4.1 Å with a force constant of ~1.2 kcal.mol^-1^Å^-2^. The metaMD bias was deposited with a Gaussian width of 0.5Å, an initial Gaussian amplitude of 0.29 kcal/mol, a deposition period of 1.0 ps and a bias factor of 12. The structures corresponding to the minimum of free energy were shown in Figure 7F-I. We find that the minimized μOR-bound DAMGO adopts a closed cytoplasmic packing that closely matches the crystallographic inactive μOR (Figure 6G). The results of these calculations were presented in Figure 7F-I & Figure S9.

In a second study, we started with the crystallographic active conformation of μOR bound to BU72 and a nanobody (PDB ID:5C1M)(*3*) to model the μOR-bound BU72. We prepared the construct by removing the all non-ligand and non-receptor molecules from the complex. Then, we reconstructed the missing residues using Swiss-pdbviewer(*2*). Subsequently, we immersed the resulting μOR-BU72 complex into a 277 POPC membrane bilayer and solvated with water and ions which led to a simulation box of 100×100×77 Å^3^, consisted of 80,000 atoms. We carried out 5000 steps of steepest descent energy minimization to relax the lipid packing around the protein construct and to relax the protein complex as well. During this process, the positions of all heavy atoms of proteins and BU72 were restrained with a force constant of ~9.6 kcal.mol^-1^Å^-2^. For POPC, we used position restraints with a force constant of ~2.4 kcal.mol^-1^Å^-2^ on just the z-coordinate of heavy atoms so that the POPC could move freely along the xy- plane to find the appropriate packing around the protein. Subsequently, we carried out a ~2ns MD simulation where we placed positional restraints on the heavy atoms with a force constant of 9.6 kcal.mol^-1^ Å^-2^. In addition, we restrained the z-coordinate of the headgroups of POPC inside the membrane with a force constant of ~2.4 kcal.mol^-1^Å^-2^. Throughout this calculation, the restraints on the protein, BU72, and POPC were gradually reduced to 0 kcal.mol^-1^Å^-2^, which prepared the construct for the further energy minimization.

To determine whether BU72 can stabilize the widely open cytoplasmic region of mouse μOR, we carried out a ~960ns metaMD simulation using Amber14(*9*) force field where we evaluate the energetics relevant to repositioning of TM6. Thus, we applied the bias forces on:

1. the distance between TM3 [the center of mass of Cα for residues 159-170] and TM6 [the center of mass of Cα for residues 272-283],
2. the distance between TM6 and TM7 [the center of mass of Cα for residues 328-339].

To expedite these calculations and limit the sampling phase space, we imposed upper and lower walls with force constant of ~24.0 kcal.mol^-1^Å^-2^ such that the distances: TM3-TM6, TM5-TM6, and TM6-TM7 vary over a range of [10.0-16.0Å],[<11Å], and [15.0-22.0Å], respectively.

During these calculations, we placed the inter-helical hydrogen bonds restraints, as mentioned above with a force constant of ~1.4 kcal.mol^-1^Å^-2^, to relax the protein structure while ensuring that the α-helical structures are stable and intact over the course of simulation.

The metaMD bias was deposited with a Gaussian width of 1.0Å, an initial Gaussian amplitude of 0.72 kcal/mol, a deposition period of 1.0 ps and a bias factor of 25. The structures corresponding to the minimum of free energy were shown in Figure 7J-L. We find that the minimized μOR-bound BU72 also adopts a closed cytoplasmic packing that closely matches the crystallographic inactive μOR (Figure 7L).

**Modeling of pre-activated human-κOR-Gi (GDP) complex.** To predict an inactive structure for the human-κOR, we used the chain A of the crystal structure of κOR (PDB ID: 4DJH)(*16*) as a template for our calculation. However, we removed the antagonist and replaced the T4 lysozyme fusion protein with the native residues 260-264, which defines ICL3. We also added the missing sequence of 301-306 to the extracellular loop 3 of κOR. Then, we subjected this modified structure to 10 cycles of simulated annealing, in which all residues on loops were heated from 50 to 600 K and cooled back to 50 K over 10 ps of simulation. We repeated this heat and quench cycle 10 times. During this process, all other residues in TM domains were fixed. We extracted the final structure of this process for further minimization. Here, we used the tight model of Gi protein described earlier to make a pre-coupled κOR-Gi (GDP) complex. However, for this study, we extended the Gαi-αN helix to include the myristoyl-Gly 2 into our model. Finally, each of modeled κOR and Gi protein was separately superimposed to the pre-coupled μOR-Gi (GDP) complex. We described all proteins, GDP, POPC membrane, water, and ions using Charmm36m(*10*) force field. We immersed the pre-coupled complex in 277 POPC membrane bilayer and solvated the system with water and ions, leading to a simulation box of 102×102×156Å^3^ with ~167,000 atoms.

We carried out 5000 steps of steepest descent energy minimization to relax the lipid around the protein construct and to relax the protein complex as well. During this process, the positions of all heavy atoms of proteins and GDP were restrained with a force constant of ~9.6 kcal.mol^-1^Å^-2^. For POPC, we used position restraints with a force constant of ~2.4 kcal.mol^-1^Å^-2^ on just the z-coordinate of heavy atoms so that the POPC could move freely along the xy- plane to find the appropriate packing around the protein. Subsequently, we carried out a ~11ns MD simulation where we placed positional restraints on the heavy atoms with a force constant of 9.6 kcal.mol^-1^ Å^-2^. In addition, we restrained the z-coordinate of the headgroups of POPC inside the membrane with a force constant of ~2.4 kcal.mol^-1^Å^-2^. Throughout this calculation, the restraints on the protein, GDP, and POPC were gradually reduced to 0 kcal.mol^-1^Å^-2^, but we restrained the salt bridge anchors: K89-D312 and D168-R32 to prepare the construct for the further energy minimization. Then, we minimized the complex by performing a ~850ns metaMD simulation using Charmm36m(*10*) to evaluate the energetics relevant to following interactions:

1. a salt bridge between the carboxylate group of F354 (C) and R156^3.50^(CZ)
2. a polar interaction between R156^3.50^(CZ)-T273^6.34^ (OG1)
3. a hydrophobic interaction between F354 (Cα) -R156^3.50^ (Cα).

We imposed upper walls on our collective variables with force constants of ~24 kcal.mol^-1^Å^-2^ at distances of 11, 13, and 10Å, respectively. For this calculation, intra-helical hydrogen bond restraints with force constants of ~1.4 kcal.mol^-1^Å^-2^ were placed on the proteins. Also, we placed harmonic restraints with a force constant ~1.2 kcal.mol^-1^Å^-2^ on the anchors.

The metaMD bias was deposited with a Gaussian width of 1Å, an initial Gaussian amplitude of 0.72 kcal/mol, a deposition period of 1.0 ps and a bias factor of 25. The results of this calculation was presented in Figure 5J-M & Figure S6A-B.

**Activation of pre-activated state of κOR -Gi protein by MP1104 (transition from Σ3’ to Σ4’*).** We followed DarwinDock procedure (*17*) to insert MP1104 to pre-activated state (Σ2) where we exploited our fully optimized MP1104 conformation in the orthosteric binding pocket of the human κOR in its fully active state (Σ4’ state). The resulting binding pocket of Σ3’ state was shown in Figure S8.

We evaluated the energetics to follow opening of Gαi subunit induced by the MP1104 binding to the pre-activated state of κOR-Gi protein by performing a ~400ns metaMD using Charmm36m(*10*) to evaluate the energetics relevant to opening the GDP binding site and structural change of AH and RAS-like domain. Thus, we followed:

1. Opening of the GDP biding site in Gαi. We considered the distance between AH (the center of mass of the Cα atoms for the residues 147-181) and Ras-like domain (the center of mass of the Cα atoms for the residues 42-59)
2. Reconfiguration of Gαi upon opening of GDP binding site. We considered the distance between AH [the center of mass of Cαs for the residues: 63-91,100-110,121-141, and 150-162] and RAS-like [the center of mass of Cαs for the residues: 220-226, 242-255, 264-280,296-310, and 319-323].

During this simulation, we imposed upper and lower walls on our collective variables with force constants of ~24 kcal.mol^-1^Å^-2^ that allowed our two collective variables to vary over a range of [16-25Å], and [31.5-41.5Å] respectively. We also placed a restraint with a force constant of ~1.2 kcal.mol^-1^Å^-2^ on the typical salt bridge between D138^3.32^ and the protonated N atom of MP1104. During this calculation, intra-helical hydrogen restraints with force constants of ~1.4 kcal.mol^-1^Å^-2^ were placed on the proteins. Also, we kept the anchors by placing harmonic restraints with a force constant ~0.6 kcal.mol^-1^Å^-2^.

Here, the metaMD bias the bias was deposited with a Gaussian width of 1 Å, an initial Gaussian amplitude of 0.72 kcal/mol, a deposition period of 1.0 ps and a bias factor of 25. The results of this calculation were presented in Figure 6B & Figure S8.

**Optimization of κOR-MP1104: Initiation of activation by Ligand-First mechanism of activation.** We followed DarwinDock procedure (*17*) to insert MP1104 to inactive state of κOR (Σ0) where we exploited our fully optimized MP1104 conformation in the orthosteric binding pocket of the human κOR in its fully active state (Σ4’ state). The resulting binding pocket was shown in Figure 8A.

To determine whether MP1104 binding to inactive κOR leads to opening the polar interaction between R156^3.50^-T273^6.34^, we performed a ~710ns metaMD simulation using Charmm36m(*10*) to assess the energetics relevant to following hydrogen bonds:

1. T273^6.34^(OG1)-R156^3.50^(NE)
2. T273^6.34^(OG1)-R156^3.50^(NH1)
3. T273^6.34^(OG1)-R156^3.50^(NH2)

To expedite this process, we imposed upper walls on our collective variables with force constants of ~24 kcal.mol^-1^Å^-2^ at distances of 8.5Å. Also, we placed a set of harmonic restraints on the inter-helical hydrogen bonds in order to relax the protein structure while ensuring that the α-helical structures are stable and intact over the course of simulation. The distance between N (i+4) and C (i) atoms of the residues in all α-helices were restrained at a distance of 4.1 Å with a force constant of ~1.2 kcal.mol^-1^Å^-2^. We also placed a restraint with a force constant of ~1.2 kcal.mol^-1^Å^-2^ at a distance of 3 Å of the typical salt bridge between D138^3.32^ and the protonated N atom of MP1104. The metaMD bias was deposited with a Gaussian width of 0.5Å, an initial Gaussian amplitude of 0.29 kcal/mol, a deposition period of 1.0 ps and a bias factor of 12. The result of this calculation was presented in Figure 8A-D & Figure S10.

**Modeling of pre-activated human-δOR-Gi (GDP) complex.** To predict the inactive structure for human-δOR, we used the crystal structure (PDB ID: 4N6H)(*18*) as a template. However, we removed the ligand and soluble cytochrome b562 from the N-terminus. Here, we used the tight model of Gi protein described earlier to make a pre-coupled δOR-Gi (GDP) complex. Subsequently, both δOR and Gi protein were separately superimposed to the pre-coupled μOR-Gi (GDP) complex. We described all proteins, GDP, POPC membrane, water, and ions using Charmm36m force field(*10*) during our free energy calculation.

We carried out 5000 steps of steepest descent energy minimization to relax the lipid packing around the protein construct and to relax the protein complex as well.

During this process, the positions of all heavy atoms of proteins and GDP were restrained with a force constant of ~9.6 kcal.mol^-1^Å^-2^. For POPC, we used position restraints with a force constant of ~2.4 kcal.mol^-1^Å^-2^ on just the z-coordinate of heavy atoms so that the POPC could move freely along the xy- plane to find the appropriate packing around the protein. Subsequently, we carried out a ~6ns MD simulation where we placed positional restraints on the heavy atoms with a force constant of 9.6 kcal.mol^-1^ Å^-2^. In addition, we restrained the z-coordinate of the headgroups of POPC inside the membrane with a force constant of ~2.4 kcal.mol^-1^Å^-2^. Throughout this calculation, the restraints on the protein, GDP, and POPC were gradually reduced to 0 kcal.mol^-1^Å^-2^, but we restrained the salt bridge anchors: K79-D312 and D158-R32 to prepare the construct for the further energy minimization. Then, we minimized the complex by performing a ~500ns metaMD simulation using Charmm36m(*10*) to evaluate the energetics relevant to following interactions:

1. the salt bridge between the carboxylate group of F354 (C) and R146^3.50^(CZ)
2. the hydrogen bond between R146^3.50^(CZ)-T260^6.34^ (OG1)
3. the hydrophobic interaction between F354 (Cα) -R146^3.50^ (Cα).

We imposed upper walls on our collective variables with force constants of ~24 kcal.mol^-1^Å^-2^ at distances of 11, 13, and 10Å, respectively. For this calculation, intra-helical hydrogen bond restraints with force constants of ~1.4 kcal.mol^-1^Å^-2^ were placed on the proteins. Also, we placed harmonic restraints with a force constant ~1.2 kcal.mol^-1^Å^-2^ on the anchors.

The metaMD bias was deposited with a Gaussian width of 1Å, an initial Gaussian amplitude of 0.72 kcal/mol, a deposition period of 1.0 ps and a bias factor of 25. The results of these calculations were presented in Figure 5N-Q & Figure S6C-D.

**Activation of pre-activated state of δOR-Gi protein by DPI-287 (transition from Σ3’ to Σ4’*).** To insert DPI-287 to the pre-activated state (Σ2), we exploited our fully optimized DPI-287 conformation in the orthosteric binding pocket of the human δOR, in its fully active state (Σ4’ state). Thus, we superimposed the Σ2 state to the fully active human δOR (Σ4’). To locate and optimize the interactions between human δOR and DPI-287, we performed 10ns (500 cycles) of simulated annealing, in which the system was first heated from 25 to 600 K over 20ps with a sequence of 25, 100, 310, 450, 600K and then sharply cooled back to 310K over 30 ps. In this calculation, we placed harmonic restraints on backbone atoms and ligand with a force constant of ~9.6 kcal.mol^-1^Å^-2^, while the side chains were free to find the optimum conformation. The resulting binding pocket for Σ3’ states was shown in Figure S8.

We evaluated the energetics to follow opening of Gαi subunit induced by the DPI-287 binding to the pre-activated state of δOR-Gi protein by performing a ~400ns metaMD using Charmm36m(*10*) to evaluate the energetics relevant to opening the GDP binding site and structural change of AH and RAS-like domain. Thus, we followed:

1. Opening of the GDP biding site in Gαi. We considered the distance between AH (the center of mass of the Cα atoms for the residues 147-181) and Ras-like domain (the center of mass of the Cα atoms for the residues 42-59)
2. Reconfiguration of Gαi upon opening of GDP binding site. We considered the distance between AH [the center of mass of Cαs for the residues: 63-91,100-110,121-141, and 150-162] and RAS-like [the center of mass of Cαs for the residues: 220-226, 242-255, 264-280,296-310, and 319-323].

During this simulation, we imposed upper and lower walls on our collective variables with force constants of ~24 kcal.mol^-1^Å^-2^ that allowed our two collective variables to vary over a range of [15-24Å], and [32-42Å] respectively. We also placed a restraint with a force constant of ~1.2 kcal.mol^-1^Å^-2^ on the hydrogen bond between D128^3.32^ (OD2) and the protonated N atom of DPI-287. During this calculation, intra-helical hydrogen restraints with force constants of ~1.4 kcal.mol^-1^Å^-2^ were placed on the proteins. Also, we kept the anchors by placing harmonic restraints with a force constant ~0.6 kcal.mol^-1^Å^-2^.

Here, the metaMD bias the bias was deposited with a Gaussian width of 1 Å, an initial Gaussian amplitude of 0.72 kcal/mol, a deposition period of 1.0 ps and a bias factor of 25. The results of these calculations were presented in Figure 6C.

**Optimization of δOR-DPI-287: Initiation of activation by Ligand-First mechanism of activation.** To insert DPI-287 to the inactive state (Σ0) of δOR, we exploited our fully optimized DPI-287 conformation in the orthosteric binding pocket of the human δOR, in its fully active state (Σ4’ state). Thus, we superimposed the Σ0 state to the fully active human δOR (Σ4’). To locate and optimize the interactions between human δOR and DPI-287, we performed 10ns (500 cycles) of simulated annealing, in which the system was first heated from 25 to 600 K over 20ps with a sequence of 25, 100, 310, 450, 600K and then sharply cooled back to 310K over 30 ps. In this calculation, we placed harmonic restraints on backbone atoms and ligand with a force constant of ~9.6 kcal.mol^-1^Å^-2^, while the side chains were free to find the optimum conformation. The resulting binding pocket for Σ3’ states was shown in Figure 8E.

To determine whether DPI-287 binding to inactive δOR leads to opening the polar interaction between R146^3.50^-T260^6.34^, we performed a ~460ns metaMD simulation using Charmm36m(*10*) to assess the energetics relevant to following hydrogen bonds:

1. T260^6.34^(OG1)-R146^3.50^(NE)
2. T260^6.34^(OG1)-R146^3.50^(NH1)
3. T260^6.34^(OG1)-R146^3.50^(NH2)

To expedite this process, we imposed upper walls on our collective variables with force constants of ~12 kcal.mol^-1^Å^-2^ at distances of 8.5 Å. Also, we placed a set of harmonic restraints on the inter-helical hydrogen bonds in order to relax the protein structure while ensuring that the α-helical structures are stable and intact over the course of simulation. The distance between N (i+4) and C (i) atoms of the residues in all α-helices were restrained at a distance of 4.1 Å with a force constant of ~1.2 kcal.mol^-1^Å^-2^. We also placed a restraint with a force constant of ~1.2 kcal.mol^-1^Å^-2^ at a distance of 3 Å of the hydrogen bond between D128^3.32^ (OD2) and the protonated N atom of DPI-287. The metaMD bias was deposited with a Gaussian width of 0.5Å, an initial Gaussian amplitude of 0.29 kcal/mol, a deposition period of 1.0 ps and a bias factor of 12. The result of this calculation was presented in Figure 8E-I & Figure S10.

**Force fields and algorithms for free energy calculations.** In simulations using Amber force field, the protein was described using Amber14(*9*) and the parameters for POPC were borrowed from LIPID14(*9*). The force field parameters for all bonded and non-bonded interactions of DAMGO, BU72, and morphine were obtained from the Generalized Amber force field(*19*) using ACPYPE(*20*) and Antechamber16(*21*). The partial charges for the ligands were assigned with the semi-empirical AM1-BCC model(*22*) as incorporated in USCF chimera(*23*). The GDP parameters were borrowed from a combination of the Amber force field and the Meagher et al. et al. study.(*24*, *25*) The TIP3P(*26*) model was used to describe the water. For simulations using the charmm36m force field, proteins, POPC, GDP, and ions were described using the Charmm36m(*10*) parameter set. Water was described using the TIP3P model. The ligand was parameterized using the ParamChem server(*27*, *28*). For the calculation using OPLS force field, we described protein(*11*), POPC(*29*, *30*), and ligands(*31*–*33*) with parameters compatible with OPLS force field.

In our well-tempered metaMD(*34*) simulations, the temperature was maintained at 310K using a velocity-rescale(*35*) thermostat with a damping constant of 1.0 ps for temperature coupling and the pressure was controlled at 1 bar using a Parrinello-Rahman barostat algorithm(*36*) with a 5.0 ps damping constant for the pressure coupling. Semi-isotropic pressure coupling was used during this calculation. The Lennard-Jones cutoff radius was 10 Å, where, the interaction was smoothly shifted to 0 after 10 Å. Periodic boundary conditions were applied to all three directions. The Particle Mesh Ewald algorithm(*12*) with a real cutoff radius of 10 Å and a grid spacing of 1.2 Å was used to calculate the long-range coulombic interactions. Compressibility of 4.5 ×10^-5^ bar^-1^ was used in the xy- plane and also the z axis, to relax the box volume. In all the above simulations**,** water OH-bonds were constrained by the SETTLE algorithm(*37*). The remaining H-bonds were constrained using the P-LINCS algorithm(*38*). All simulations were carried out using GROMACS(*39*) and metaMD calculations were done using PLUMED-2(*40*).

**2. Figures**

**

**

**Fig. S1.** Evaluation of the strength for the anchors between Gi protein and each of three ICLs of mouse μOR. Variation of the average free energy difference with time to monitor the metaMD convergence. The average free energy difference was calculated every 500ps between two local minima: (A) a salt bridge between K98^ICL1^(NZ)-D312^Gβ^(CG) (shown by purple), and a salt bridge between D177^ICL2^(CG)-R32^G^*^α^*^i^ (CZ) (shown by green); (B) a salt bridge between K271^6.26^(NZ)-E318^G^*^α^*^i^ (CD) (shown by purple); and (D) a salt bridge between R263^ICL3^ (CZ)-E318^G^*^α^*^i^ (CD) (shown by green).

**Fig. S2.** **Structure of the mouse μOR–Gi Protein complex derived from MD simulations.** Extensive polar interactions (salt bridges and hydrogen bonds) between Gi protein and mouse μOR.





**Fig. S3. Gi protein binds the human μOR by forming ionic anchors to ICL1, ICL2, and the cytoplasmic end of TM6, despite the choice of force fields.** (A) Structure of the human μOR–Gi Protein complex derived from a 1 μs of MD simulation using Charmm36m force field. B) Direct ionic interactions from Gβ subunit to the ICL1, C) an anchor from G*α*i subunit to the ICL2, D) a network of polar interactions between ICL2 and Gαi-α5 helix, and E) anchoring from Gαi subunit to the ICL3 and cytosolic end of TM6. F) RMSD variation of the complex with time. (G), (H), & (I) Variation of the salt bridge anchors between Gi protein and μOR with time. J) Structure of the human μOR–Gi Protein complex derived from a 1 μs of MD simulation using the OPLS force field. K) Direct ionic interactions from Gβi subunit to the ICL1 and H8. L) An anchor from G*α*i *subunit* to the ICL2 and M) A network of polar interactions between ICL2 and Gαi-α5 helix. N) Anchoring from Gαi subunit to the ICL3 and cytosolic end of TM6. O) RMSD variation of the complex with time. Here, RMSD was calculated for backbone atoms and the whole structures over the course of simulation and each trajectory was compared to the final snapshot. (P), (Q), & (R) Variation of the salt bridge anchors between Gi protein and μOR with time.





**Fig. S4.** Evaluation of the energetics relevant to the formation of pre-activated state between Gi protein and μOR. MetaMD free energy: A) a hydrophobic interaction between R167^3.50^(Cα)-F354 (Cα), D) a hydrophobic interaction between R167^3.50^(Cα)-T281^6.34^ (Cα). The average free energy difference was calculated every 500ps between two local minima: (B) a salt bridge between R167^3.50^(CZ)-F354 (C), (C) a hydrophobic interaction between R167^3.50^(Cα)-F354 (Cα), and (E) a polar interaction between R167^3.50^(CZ)-T281^6.34^ (OG1).





**Fig. S5.** Evaluation of the energetics relevant to the formation of pre-activated state between the Gα-α5 peptide and μOR. MetaMD free energy: A) a hydrophobic interaction between R167^3.50^(Cα)-F354 (Cα), B) a polar interaction between R167^3.50^(CZ)-T281^6.34^ (OG1). The average free energy difference was calculated every 500ps between two local minima for each of : a salt bridge between R167^3.50^(CZ)-F354 (C), a hydrophobic interaction between R167^3.50^(Cα)-F354 (Cα), and a polar interaction between R167^3.50^(CZ)-T281^6.34^ (OG1).





**Fig. S6.** Evaluation of the energetics relevant to the formation of pre-activated state between Gi protein and either of κOR or δOR. MetaMD affinity free energy profile: (A) a hydrophobic interaction between κOR-Gi: R156^3.50^(Cα)- F354 (Cα), (C) a hydrophobic interaction between δOR-Gi: R146^3.50^(Cα)- F354 (Cα). The average free energy difference was calculated every 500ps between two local minima for each of: (B) κOR-Gi: a salt bridge between F354- R156^3.50^ (purple), a hydrophobic interaction between R156^3.50^(Cα)- F354(Cα) (blue), and breaking the polar interaction between R156^3.50^(CZ)- T273^6.34^(OG1) (green), (D) δOR-Gi: a salt bridge interaction between F354-R146^3.50^ (purple), a hydrophobic interaction between R156^3.50^(Cα)- F354(Cα) (blue), and breaking the polar interaction between R146^3.50^- T260^6.34^ (green).





**Fig. S7.** A candidate structure through the formation of the Gi protein-δOR pre-activated complex (Σ2) suggests that the carboxylate group of F354 makes a salt bridge with the R146^3.50^ just as for μOR and κOR, leading to breaking the polar interaction between R146^3.50^ and T260^6.34^.





**Fig. S8.** The Orthosteric binding pocket of agonist in the Σ3’ of opioid receptors-Gi protein complex. The hydrophobic residues were not shown for the sake of clarity.





**Fig. S9. Agonists cannot not stabilize the active conformation of μOR.** MetaMD affinity free energy profile: **(A)** the distance between TM6 [the center of mass of Cα for residues 274-285] and TM7 [the center of mass of Cα for residues 330-341], **(B)** the distance between TM5 [the center of mass of Cα for residues] and TM6 in the S1 state of μOR-morphine. **(C)** The average free energy difference was calculated every 500ps between two local minima for each of: TM3 [the center of mass of Cα for residues: 161-172]-TM6 (purple), TM5-TM6 (orange), and TM6-TM7 (green). **(D)** MetaMD affinity free energy profile for a polar interaction between R167^3.50^(NE)-T281^6.34^(OG1). **(E)** The average free energy difference was calculated every 500ps between two local minima for each of: a hydrogen bond between R167^3.50^(NH1)-T281^6.34^(OG1), a hydrogen bond between R167^3.50^(NH2)-T281^6.34^(OG1), and a polar interaction between R167^3.50^(NE)-T281^6.34^(OG1). **(F)** MetaMD affinity free energy profile for the distance between TM6 [the center of mass of Cα for residues 272-283] and TM7 [the center of mass of Cα for residues 328-339] in the S1 state of mouse μOR-DAMGO. (**G**) The average free energy difference was calculated every 500ps between two local minima for each of: TM3 [the center of mass of Cα for residues: 159-170]-TM6 (purple), and TM6-TM7 (green). **(H)** MetaMD affinity free energy profile for a polar interaction between R165^3.50^(NH2)-T279^6.34^(OG1). **(I)** The average free energy difference was calculated every 500ps between two local minima for each of: a hydrogen bond between R165^3.50^(NH1)-T279^6.34^(OG1), a hydrogen bond between R165^3.50^(NE)-T279^6.34^(OG1), and a polar interaction between R165^3.50^(NH2)-T279^6.34^(OG1). **(J)** MetaMD affinity free energy profile for the distance between TM6 and in the S1 state of mouse μOR-BU72. (**K**) The average free energy difference was calculated every 500ps between two local minima for each of: TM3-TM6 (purple), and TM6-TM7 (green).





**Fig. S10. The energetically optimized S1 state of κOR-MP1104 and δOR-DPI-287.** (A) MetaMD affinity free energy profile for a polar interaction between R156^3.50^(NH1)-T273^6.34^(OG1) in the S1 state of κOR-MP1104. (B) The average free energy difference was calculated every 500ps between two local minima for each of: a polar interaction between R156^3.50^(NH1)-T273^6.34^(OG1), a hydrogen bond between R156^3.50^(NE)-T273^6.34^(OG1), and hydrogen bond between R156^3.50^(NH2)-T273^6.34^(OG1) in the S1 state of κOR-MP1104. (C) The average free energy difference was calculated every 500ps between two local minima for each of: a broken polar interaction between R146^3.50^(NH1)-T260^6.34^(OG1), a broken polar interaction between R146^3.50^(NE)-T260^6.34^(OG1), and a broken polar interaction between R146^3.50^(NH2)-T260^6.34^(OG1) in the S1 state of δOR-DPI-287.





**Fig. S11.** **Gαi subunit interactions with GDP in pre-activated state (Σ2).** GDP is stabilized through several polar interactions with both Gαi Ras-like and α-helical (AH) domain.





**Fig. S12.** Evaluation of the RMSD for: A) Gαi-α5 peptide, B) μOR, C) μOR-Gαi-α5 peptide during the formation of pre-activated state between the Gα-α5 peptide and μOR.





**Fig. S13.** **The** **second replicate of metaMD calculations to assess the formation of pre-activated complex between Gi protein and human μOR prior to ligand binding**. A) The structure of the pre-coupled state (Σ1 state), comprising the inactive human μOR and inactive Gi protein-bound GDP (Tight); the Gi protein binds to the inactive μOR by forming salt bridge anchors to ICL1, ICL2, and ICL3. In this calculation, we included a Na^+^ next to D116^2.50^ to balance the charge. B) MetaMD free energy profile for the salt bridge between the F354 (C) and R167^3.50^ (CZ). C) The structure of the pre-activated state (Σ2) showing open μOR (broken salt bridge interaction R167^3.50^ -T281^6.34^), and new salt bridge between F354-R167^3.50^. (B) The weighted averages and the standard deviations were calculated for the converged period between the initial configuration [pre-coupled Σ1 state denoted “*i”*] and the final configuration [pre-activated Σ2 state, denoted “*f*”] after metaMD calculations.





**Fig. S14.** Binding of agonist to μOR cannot open the TM3-6 hydrogen bond in the absence of Gi protein. (A*)* The minimized structure for the human μOR-morphine complex, in the absence of Gi protein or a mimetic Gi protein nanobody. Figures B and C shows that the agonist alone cannot open up the cytoplasmic region of μOR, with its hydrogen bond between R^3.50^ and T^6.34^. In these calculations, we included the Na^+^ interacting with D116^2.50^ to form a neutral complex. MetaMD free energy of (B) the interaction between R167^3.50^(NE)-T281^6.34^(OG1), and (C) the interaction between R167^3.50^ (NH2)-T281^6.34^(OG1). (D) The weighted averages and the standard deviations were calculated for the converged period between the initial configuration, “*i”,*  and the final conformation, “*f*”, after metaMD calculations.





**Fig. S15.** Comparisons of the cytoplasmic region of the inactive state and the pre-activated state for (A) μOR, (B) κOR, and (C) δOR. All RMSDs were calculated for the Cα atoms on the 7TM core.





**Fig. S16. Agonist binding is the main driver of activation for the μOR and Gi protein. (A)**  The optimized unliganded μOR (apo) coupled to inactive Gi protein-bound GDP. **(B)** MetaMD free energy of the distance between the AH domain (the center of mass of the Cα atoms for the residues 147-181) and the Ras-like domain (the center of mass of the Cα atoms for the residues 42-59), which define the GDP binding site. We see that without the agonist it is uphill 30 kcal/mol to open up the Gα to release GDP (constitutive activity). **(C)** The variation of the free energy difference with time was calculated to monitor the free energy convergence. The weighted averages and the standard deviations were calculated for the converged period.





**Fig. S17. Activation of the Gi protein is induced by agonist binding to the opioid receptors-Gi Protein complex. (a)** the optimized unliganded-μOR (apo) in the activated state, coupled to inactive Gi protein-bound GDP. To model the unliganded-μOR (apo) in the activated state, we started with our optimized morphine-μOR-Gi protein [nucleotide-free] complex (state Σ4’), and then eliminated the morphine and the Gi protein from the complex. **(B)** MetaMD free energy of the distance between the AH domain (the center of mass of the Cα atoms for the residues 147-181) and the Ras-like domain (the center of mass of the Cα atoms for the residues 42-59) that define the GDP binding site. **(C)** The variation of the free energy difference with time was calculated to monitor the free energy convergence. The weighted averages and the standard deviations were calculated for the converged period.
